# Cannabidiol Use in Inflammatory Bowel Disease: Insights From a Gastroenterology Outpatient Population

**DOI:** 10.1002/jgh3.70402

**Published:** 2026-04-07

**Authors:** Karina Fatakhova, Muhammad Jahanzaib Khan, Royce Perera, Sapphire Perera, Benjamin Glazebnik, Lindsey Donnelly, Pratik Patel, Emily Glazer, Greg Haggerty, Hassam Ali, Ramona Rajapakse

**Affiliations:** ^1^ Division of Gastroenterology and Hepatology Northwell, Mather Hospital and Zucker School of Medicine at Hofstra University Port Jefferson New York USA; ^2^ Cornell University Ithaca New York USA; ^3^ New York University New York New York USA; ^4^ Vassar College Poughkeepsie New York USA; ^5^ Division of Gastroenterology and Hepatology University of South Florida Tampa Florida USA; ^6^ Division of Gastroenterology and Hepatology East Carolina University Greenville North Carolina USA

**Keywords:** Cannabidiol, complementary therapies, inflammatory bowel diseases, patient reported outcomes, surveys and questionnaires

## Abstract

Cannabidiol (CBD) is increasingly used for symptom relief in chronic conditions, yet its role in inflammatory bowel disease (IBD) remains underexplored. We surveyed 229 IBD patients and found that 10.5% used CBD, primarily for anxiety (54.2%), insomnia (41.7%), and pain (41.7%). Among users, 87.5% perceived benefit. Most users were female, and one‐third were on biologics. These findings highlight the growing interest in alternative therapies and the need for further research on CBD's safety, efficacy, and clinical guidance in IBD care.

## 1 Introduction

Cannabidiol (CBD), a cannabinoid compound found in cannabis, from the plant *Cannabis sativa*, has garnered increasing interest within the medical community in recent years. This hemp plant has been used for medicinal purposes for over 4000 years in China [1]. Unlike the more commonly known compound, tetrahydrocannabinol, also found in cannabis, it is nonintoxicating and does not have any psychoactive effects. CBD is easily obtainable through a variety of different retailers. It is commonly mixed in an oil or gummy form and is available in different strengths and flavors. CBD has been shown to have anxiolytic, anti‐inflammatory, antiemetic, and antipsychotic properties [2, 3]. Considering CBD's recognized anti‐inflammatory characteristics, it can be inferred that it could potentially be beneficial to patients with Inflammatory Bowel Disease (IBD).

Despite extensive research on the impact of cannabis on IBD patients, there is a scarcity of studies examining the utilization and impacts of CBD within this demographic. International surveys report cannabis or CBD use rates in IBD ranging from 15% to 30% [4, 5, 6], with patients often citing pain, sleep, and mood disturbances as the most common reasons. U.S. data are limited, with few outpatient cohorts systematically surveyed.

The aim of our survey‐based study is to determine the prevalence, reasons for use, and perceived benefits of CBD among IBD patients in a U.S. outpatient gastroenterology population. By doing so, we provide updated prevalence estimates, explore patient‐reported motivations, and contextualize findings in this patient population.

## 2 Methods

This was a cross‐sectional survey‐based study conducted over a 2‐week period from June 1 to June 16, 2022. A total of 229 adult patients (aged ≥ 18 years) with a confirmed diagnosis of IBD, including both Crohn's disease and ulcerative colitis, were recruited from two outpatient gastroenterology clinics in Suffolk County, New York. The study was approved by the local institutional review board, and verbal informed consent was obtained from all participants prior to enrollment. Eligible patients were identified through clinic appointment schedules and invited to participate either during their in‐person clinic visit or via telephone outreach. Participants were informed that the survey was anonymous, voluntary, and would not affect their medical care. The survey was developed de novo by the study investigators and consisted of 13 multiple‐choice, short, close‐ended questions. It included sections on demographic characteristics (age, sex), IBD‐related clinical history (type of IBD, disease duration, prior surgery, use of biologics), personal history of malignancy, and CBD use. Questions on CBD addressed current or prior use, form of administration (oil, gummies, etc.), indications for use (e.g., anxiety, pain, insomnia), and perceived benefit. Perceived benefit was assessed using a binary yes/no response. Nonresponder data were not systematically captured. Survey responses were recorded directly by the patients on paper during the office visit or by the provider during telephone interviews, then transcribed into a secure, anonymized database. Descriptive statistics were used to analyze the frequency and distribution of responses. All data were stored and analyzed using Excel and figures were generated using Generative Pre‐trained Transformer (GPT‐5).

## 3 Results

**FIGURE 1:**
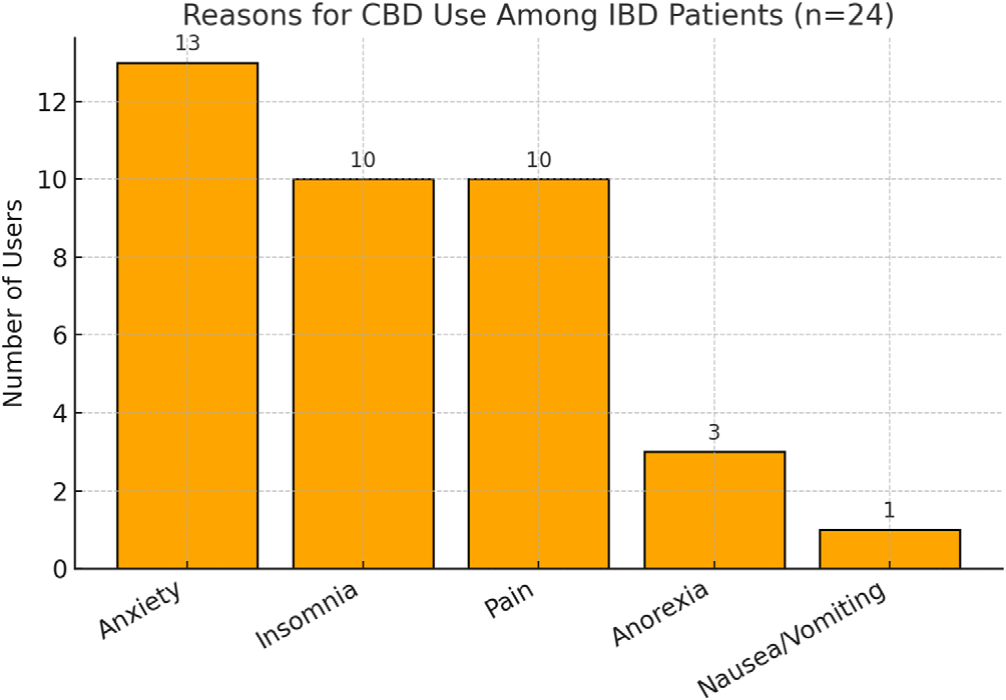
Reasons for CBD use in IBD patients.

Of the 229 patients surveyed, 24 (10.5%, 95% CI: 7.1%–15.1%) reported current or prior use of CBD products, most commonly in the form of oils or gummies. As shown in Table 1, the median age of CBD users was 49.5 years (IQR: 32.5–61.0), slightly lower than non‐users (median: 54.0, IQR: 40.0–66.0), though not statistically significant (*p* = 0.178). CBD users were more likely to have a history of Crohn's disease (58.3%) compared to non‐users (50.7%), and less likely to report ulcerative colitis (50.0% vs. 55.1%), although these differences were not significant. Use of biologic therapy was similar between groups (33.3% vs. 33.7%), while prior IBD‐related surgery was numerically higher among CBD users (37.5% vs. 22.9%, *p* = 0.134). The most frequently reported reasons for CBD use included anxiety (54.2%, 95% CI: 35.1%–72.1%), insomnia (41.7%, 95% CI: 24.5%–61.2%), and pain (41.7%, 95% CI: 24.5%–61.2%), followed by anorexia (12.5%, 95% CI: 4.3%–31.0%) and nausea/vomiting (4.2%, 95% CI: 0.7%–20.2%), as shown in Table S1 and visualized in Figure 1. Multiple reasons could be selected by each respondent. Among CBD users, 87.5% (21/24) reported that CBD use helped with symptom management. Table S1 displays the frequency and 95% confidence intervals for each reported reason for CBD use among the 24 CBD‐using patients. Figure S1 provides the survey instrument used to assess CBD product use among IBD patients.

**TABLE 1:** Baseline characteristics of IBD patients by CBD use.

Variable and category	CBD users (*n* = 24)	Non‐users (*n* = 205)	Total (*n* = 229)	*p*
Age (years)	49.5 (32.5–61.0)	54.0 (40.0–66.0)	54.0 (39.0–64.0)	0.178
Crohn's disease	14 (58.3%)	104 (50.7%)	118 (51.5%)	0.525
Ulcerative colitis	12 (50.0%)	113 (55.1%)	125 (54.6%)	0.669
On biologic therapy	8 (33.3%)	69 (33.7%)	77 (33.6%)	1.000
History of IBD surgery	9 (37.5%)	47 (22.9%)	56 (24.5%)	0.134

## 4 Discussion

In this cross‐sectional survey, 10.5% of IBD patients reported CBD use, primarily for anxiety, insomnia, and pain. CBD users were more likely to be female and to have Crohn's disease. One‐third were on biologics, and over one‐third had undergone IBD‐related surgery. While CBD use among IBD patients appears lower than that reported in other chronic disease populations such as arthritis or chronic pain, where rates of 20%–30% have been observed [7], this comparison should be interpreted cautiously due to differences in study design and populations.

CBD has demonstrated anti‐inflammatory and immunomodulatory effects, partly via inhibition of FAAH and activation of PPARγ, which enhance endocannabinoid tone and reduce proinflammatory signaling [8, 9]. These properties have shown therapeutic potential in colitis models and other inflammatory conditions [10]. In contrast, our findings reflect self‐reported symptom relief. Although a substantial 87.5% reported symptomatic benefit, this should be interpreted cautiously due to potential placebo effect, self‐selection bias, and lack of objective outcomes. Therefore, the observed real‐world benefits may relate more to symptomatic modulation than direct anti‐inflammatory effects.

Anxiety was the most frequently reported indication (54.2%). Prior studies have identified elevated anxiety in IBD patients, often linked to unpredictable disease flares and chronic pain. CBD may exert anxiolytic effects through serotonergic pathways and 5HT1A receptor modulation [11, 12]. Frane et al. similarly found improvements in anxiety, sleep, and pain among arthritis patients using CBD [7].

Pain, reported by 41.7% of users, is a complex and common symptom in IBD. CBD may reduce inflammation‐related abdominal pain and visceral hypersensitivity, though human data remain limited [13]. In our cohort, insomnia was also a leading reason for use. While evidence on CBD's effect on sleep remains mixed, its anxiolytic impact may promote sleep indirectly [14]. Notably, self‐reported reasons for CBD use were more commonly related to neuropsychiatric symptoms, such as anxiety and insomnia, than to gastrointestinal symptoms, suggesting that patients with IBD may be seeking CBD primarily for mental health and sleep‐related concerns rather than for direct gastrointestinal symptom relief.

CBD is currently not recommended in the U.S. IBD management guidelines, and controlled studies are needed to establish its clinical utility. Clinicians should remain aware of variability in formulations, quality, and dosing of commercially available products. In our cohort, no adverse effects were reported, though prior studies have described fatigue, diarrhea, and potential drug–drug interactions [15].

Limitations include self‐reported data rather than validated instruments, small sample size, and lack of dose/formulation standardization. We also did not adjust for multiple comparisons or potential confounders such as age, sex, or disease subtype given the limited number of CBD users, which may restrict the generalizability of our findings.

In this cross‐sectional survey of a U.S. outpatient IBD population, approximately one in ten patients reported CBD use, most commonly for anxiety, insomnia, and pain, with a high proportion perceiving symptomatic benefit. CBD use appeared more frequent among patients with Crohn's disease and those with prior surgical history, although differences were not statistically significant. These findings suggest that patients with IBD may be increasingly turning to CBD primarily for neuropsychiatric and symptom‐related relief rather than direct gastrointestinal disease control. Given the absence of guideline recommendations and the variability in product quality, dosing, and regulation, clinicians should proactively inquire about CBD use and counsel patients accordingly.

## Funding

The authors have nothing to report.

## Conflicts of Interest

The authors declare no conflicts of interest.

## Supporting information


**Table S1:** Reasons for CBD use among IBD patients (*n* = 24).
**Figure S1:** Survey: Use of CBD products among IBD patients in Long Island, NY.

## Data Availability

The data that support the findings of this study are available from the corresponding author upon reasonable request.
