# Supplementary material for: Cannabidiol Use in Inflammatory Bowel Disease: Insights From a Gastroenterology Outpatient Population
Source: JGH Open. 2026 Apr 7;10(4):e70402. doi: 10.1002/jgh3.70402 (PMC13056695; doi:10.1002/jgh3.70402)

Supplementary Table 1. Reasons for CBD Use Among IBD Patients (n=24)

| **Reason** | **n** | **N** | **Proportion (%)** | **95% CI** |
| --- | --- | --- | --- | --- |
| Anxiety | 13 | 24 | 54.2% | 35.1–72.1% |
| Insomnia | 10 | 24 | 41.7% | 24.5–61.2% |
| Pain | 10 | 24 | 41.7% | 24.5–61.2% |
| Anorexia | 3 | 24 | 12.5% | 4.3–31.0% |
| Nausea/Vomiting | 1 | 24 | 4.2% | 0.7–20.2% |

Supplementary Figure 1: Survey: Use of CBD products amongst IBD patients in Long Island, NY


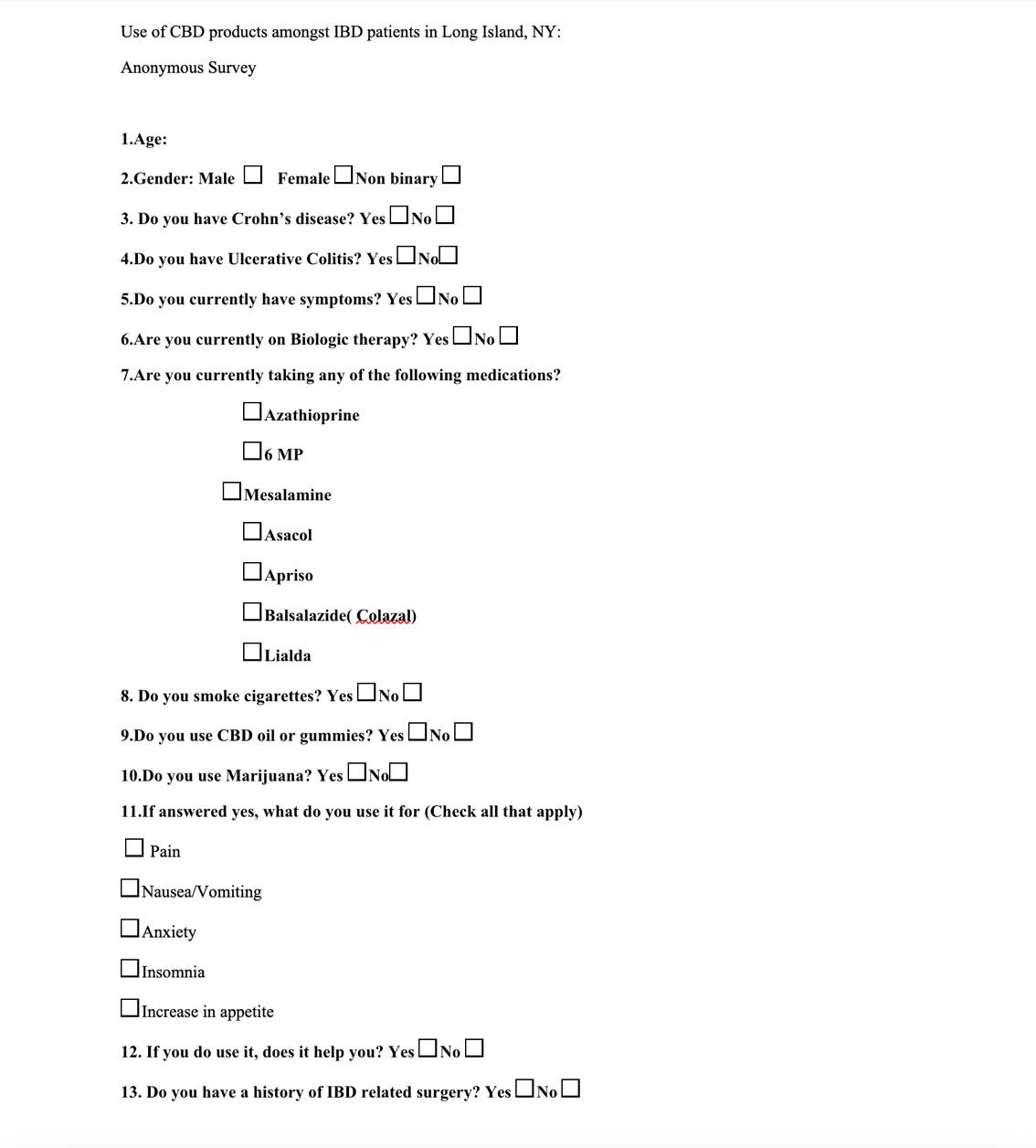

Supplement: Supplementary file 1 — Table S1: Reasons for CBD use among IBD patients (n = 24). Figure S1: Survey: Use of CBD products among IBD patients in Long Island, NY. [file JGH3-10-e70402-s001.docx]
